# Supplementary material for: Impact of BNT162b first vaccination on the immune transcriptome of elderly patients infected with the B.1.351 SARS-CoV-2 variant
Source: medRxiv. 2021 May 14:2021.05.11.21256862. Preprint. [Version 1] doi: 10.1101/2021.05.11.21256862 (PMC8132253; doi:10.1101/2021.05.11.21256862)
Supplement: 1 — Supplementary Figure 1. Upregulation of Interferon stimulated genes (ISGs), innate antiviral immunity and COVID-19-SARS-CoV-2 infection genes in vaccinated COVID-19 patients. a. Heatmap showing the significant enriched genes related to immune responses and SARS-CoV-2 virus infection. b. mRNA levels of JAK/STAT signaling components, STAT1, STAT2 and SOCS3, measured by RNA-seq were presented by bar graphs. S: sample [file NIHPP2021.05.11.21256862V1-supplement-1.pdf]

medRxiv preprint doi: https://doi.org/10.1101/2021.05.11.21256862; this version posted May 14, 2021. The copyright holder for this preprint (which was not certified by peer review) is the author/funder, who has granted medRxiv a license to display the preprint in perpetuity. This article is a US Government work. It is not subject to copyright under 17 USC 105 and is also made available for use under a CC0 license.

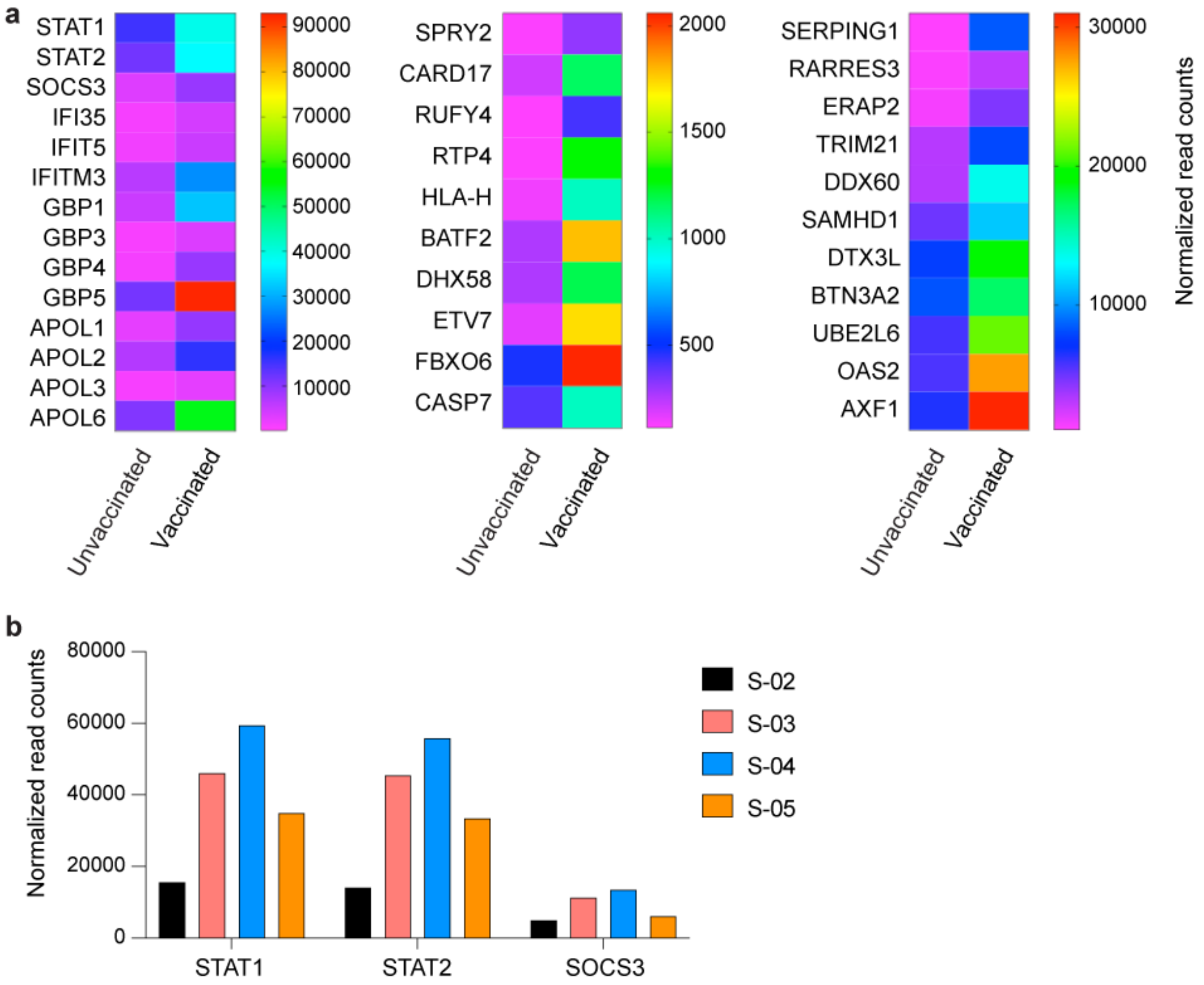

Supplementary Figure 1. Upregulation of Interferon stimulated genes (ISGs), innate antiviral immunity and COVID-19-SARS-CoV-2 infection genes in vaccinated COVID-19 patients. a. Heatmap showing the significant enriched genes related to immune responses and SARS-CoV-2 virus infection. **b.** mRNA levels of JAK/STAT signaling components, STAT1, STAT2 and SOCS3, measured by RNA-seq were presented by bar graphs. S: sample
